# Red and Processed Meat Consumption: What's at Stake?

**DOI:** 10.1093/jn/nxac036

**Published:** 2022-03-30

**Authors:** Kathryn E Bradbury

**Affiliations:** National Institute for Health Innovation, School of Population Health, University of Auckland, Auckland, New Zealand

See corresponding article on page 1254.

The World Cancer Research Fund (WCRF) and the International Agency for Research on Cancer have concluded that the consumption of unprocessed red meat probably increases the risk for colorectal cancer and that the consumption of processed meat causes colorectal cancer (1, 2). These expert bodies have made their judgments based largely on the results of meta-analyses of prospective cohort studies, most of which consisted of predominantly white participants (1, 3). Indeed, most of the nutritional epidemiology literature on various diet–disease relations comes from cohort studies in predominantly white populations, mostly from Europe and North America. The investigation of the relation between meat and colorectal cancer in the Black Women's Health Study by Yiannakou et al. (4), in this issue of the *Journal of Nutrition*, is therefore a welcome addition to the body of evidence.

In 1995, >50,000 women were recruited into the Black Women's Health Study, which was set up to investigate risk factors for disease in US black women. Diet was assessed via Food Frequency Questionnaire (FFQ) at baseline, and women have been followed for >20 y, during which time 564 incident cases of colorectal cancer occurred (4). Diet was reassessed in 2001, and this second measurement was used in the analysis by Yiannakou et al. (4) to reduce the impact of measurement error and true changes in intake over time. This is important as relying only on baseline measures of diet (or any exposure) tends to bias associations with disease toward the null (5).

The more recent of the expert body judgments, the 2018 WCRF report (1), was based on a meta-analysis of cohort studies from Europe (5 cohorts for the red meat analysis, 4 for processed meat), the United States (1 cohort for red meat—the Multiethnic Cohort Study, 4 for processed meat, including the Multiethnic Cohort Study), Australia (1 cohort for red meat, 1 for processed meat), and Asia (1 cohort for red meat, 1 for processed meat) (1). Establishing additional cohort studies in areas that are currently underrepresented in the global evidence will allow a wider range of dietary exposures and disease outcomes to be studied (6).

In the current analysis of the Black Women's Health Study, a 100-g/d higher intake of (unprocessed) red meat, about 1 small steak, was associated with a 33% (95% CI: 3%, 71%) higher risk of colorectal cancer (4). The point estimate is somewhat higher—albeit with a fairly wide confidence interval—than the summary estimate from the meta-analysis used to inform the WCRF judgment [per 100-g/d higher intake of red meat: 12% (95% CI: 6%, 25%) higher risk of colorectal cancer, *n* = 6662 cases of colorectal cancer] (1). The slightly stronger observed association in the Black Women's Health Study could be due in part to using remeasured intakes (most of the studies in the meta-analysis used only a single measure of diet at baseline) in the analysis. Alternatively, given the much smaller number of colorectal cancer cases in a single study, the stronger observed association in the Black Women's Health Study could simply be due to chance.

For processed meat, the results from the Black Women's Health Study were null but with wide confidence intervals that encompass the summary estimate from the WCRF meta-analysis [Black Women's Health Study: per 50-g/d higher intake, ∼2 rashers of bacon: 1.02 (95% CI: 0.83, 1.26) (4); WCRF per 50-g/d higher intake: 1.16 (95% CI: 1.08, 1.26), *n* = 10,738 cases] (1).

In the Black Women's Health Study, women who consumed greater amounts of meat were on average younger but in other ways were more likely to be at higher risk of developing colorectal cancer—they had a higher BMI on average, were more likely to smoke cigarettes and drink alcohol, and were less likely to do vigorous exercise (4). Similar to other cohort studies, there is no doubt that confounding by these other lifestyle factors is important, but the extent to which the fully adjusted associations are still influenced by residual confounding is not well understood.

In line with the results in the current study, the biological plausibility is stronger for red meat, as a rich source of heme iron, which leads to the formation of carcinogenic *N*-nitroso compounds in the gut (2). In terms of advancing our understanding of the underlying etiology underpinning the observed associations between red and processed meat and colorectal cancer risk, as well as further mechanistic research, it may be useful for prospective cohorts to differentiate between specific exposures related to meat consumption that may be responsible—heme iron, nitrates and nitrites, and the mutagenic heterocyclic amines and polycyclic aromatic hydrocarbons that are formed when cooking meat at high temperatures. This has been done in the US NIH–AARP cohort (7); however, in most existing cohort studies, additional data collection from participants would be needed to capture meat cooking methods and extent of cooking (meat doneness) and thus enable participants to be ranked by exposure to mutagens.

As well as any effects on health, the consumption of meat has a significant environmental impact, with studies indicating diets higher in meat and other animal-source foods produce greater greenhouse gas emissions than diets lower in animal-source foods (8). Meat from ruminant animals especially contributes to greenhouse gas emissions, due to enteric fermentation that emits large amounts of methane—a particularly potent, although relatively short-lived, greenhouse gas (8, 9). Meat production also uses significant amounts of freshwater and leads to loss of biodiversity through land conversion to agriculture. In addition, widespread use of antibiotics during production contributes to the emergence of antimicrobial-resistant bacteria (9).

The global methane pledge recently launched at the recent UN Climate Change Global Conference 2021 sets out to reduce methane emissions by 30% by 2030 (10). Signed by >100 countries, the pledge will be crucial in helping keep global warming to within 1.5°C of preindustrial levels—the target set out in the Paris Agreement (10). Action in the agriculture sector will be critical to achieving a 30% reduction in methane emissions. Governments need to urgently overcome their inertia and give serious consideration to policies that will shift the current agricultural incentives that focus on increasing productivity at the expense of environmental impact (11) and have led to a reliance and predominance of the production of products from ruminant animals.

Colorectal cancer is the third most common cancer worldwide, with 1.9 million new cases in 2020 (12). Furthermore, given that around a third of global greenhouse gas emissions are attributable to the food system, including a considerable contribution from methane from ruminant animals (13), failing to reduce global meat production and consumption is a high-stakes approach: both for the health of the population and the planet.

## Notes

Supported by a Sir Charles Hercus Health Research Fellowship from the Health Research Council of New Zealand (grant number 19/110).

Author disclosures: The author reports no conflicts of interest.
